# Laboratory features and pharmacological management of early and late-onset primary Sjögren’s syndrome

**DOI:** 10.1007/s00296-024-05626-0

**Published:** 2024-06-05

**Authors:** Rimah A. Saleem, Majed Ramadan, Yasmin Elshaaer, Hatouf Sukkarieh, Rasha Alissa, Noara Alhusseini, Hani Tamim, Awad Alshahrani, Hesham Almaimoni

**Affiliations:** 1 College of Medicine, Alfaisal University, P.O. Box 50927, Riyadh, 11533 Saudi Arabia; 2 King Abdullah International Medical Research Center (KAIMRC), Riyadh, Saudi Arabia; 3 Department of Internal Medicine and Clinical Research Institute, American University of Beirut Medical Center, Beirut, Lebanon; 4 College of Medicine, King Saud Bin Abdulaziz University for Health Sciences, Riyadh, Saudi Arabia

**Keywords:** Sjögren’s syndrome, Blood profile, Vitamins, Hydroxychloroquine

## Abstract

**Background:**

Primary Sjögren’s Syndrome (pSS) is a systemic chronic autoimmune disorder that contributes to dry mouth (xerostomia) and eyes (xerophthalmia). It mainly affects females between 40 and 60 years old. So far, there is no treatment to cure SS; however, there is a list of medications that can ameliorate the symptoms. In addition, there has been no single test until now to detect pSS, but clinical and immunological investigations are applied as diagnostic tools. Therefore, this study aimed to explore the characteristics of pSS in Saudi patients based on the onset of the disease through laboratory findings and pharmaceutical management.

**Methodology:**

This retrospective study examined diagnosed patients with pSS between 2018 and 2023 from the National Guard Hospital, Saudi Arabia. Data of pSS patients was categorized into two groups: early (under 40 years old) and late-onset (40 years old and above). Data on demographic information, mortality rate, and blood tests such as complete blood count (CBC), creatinine, erythrocyte sedimentation rate (ESR), and vitamin levels, in addition to prescribed medications, were collected from the patient’s medical record. Chi-square and t-tests were mainly used, and statistical significance was determined at a *P*-value less than 0.05.

**Results:**

A total of 453 patients were included in the study, where the early-onset group comprised 136 and the late-onset group comprised 317 patients. The mean age of the early and late onset was 34.2 and 60.4, respectively. ESR was significantly higher in the early (46.3 mm/hr) and late-onset (49.8 mm/hr). The most common medication used by all pSS patients was hydroxychloroquine. However, artificial tears were mainly observed in the late-onset group. Other medications, such as pilocarpine, methotrexate, and azathioprine, were prescribed to pSS patients to a lesser extent.

**Conclusion:**

This study suggests that the onset of pSS could occur even before the age of 40 among Saudi citizens. Notably, elevated ESR levels appeared to be a feature of pSS, which was consistent with other previous findings. The variability of some medications between early-onset and late-onset pSS may indicate disease progression. However, further investigations are required to confirm this observation.

## Introduction

Sjögren’s Syndrome (SS) is a systemic chronic autoimmune disorder that primarily affects the salivary gland alongside the lacrimal glands. The immune-mediated damage of SS results in dry mouth (xerostomia) and eyes (xerophthalmia). Dryness of mucosal surfaces, including airways, digestive tract, and vagina, may also be observed, which is clinically referred to as the sicca syndrome [1]. Nevertheless, symptoms may appear years before diagnosis [2–5]. It is classified into primary (pSS) or secondary SS based on its association with other autoimmune diseases like rheumatoid arthritis (RA) or systemic lupus erythematosus (SLE) [1, 6]. It mainly affects females compared to males at a ratio of 9:1, respectively, and can occur at any age, yet the peak prevalence is approximately 50 years old [2, 5]. Although the etiology of this disorder is not fully understood, it has been suggested that the Epstein–Barr virus and genetic factors may play a role in the pathogenesis of SS [1, 7].

According to numerous epidemiological studies, which have been summarized by Narváez et al., the incidence of pSS is between 0.1 and 3% [8], with the highest occurrence rate being 6-11.8 per 100,000 persons reported in Asia [9]. A key feature of pSS pathogenesis includes B-cell hyperactivity alongside the upregulation of proinflammatory cytokines [10, 11]. The clinical features of pSS are variable, yet fatigue accounts for 70–80% of pSS cases. In addition, extra-glandular manifestations, such as the liver, lungs, and renal systems, may be involved [1, 12]. Currently, pSS is treated by different medications such as saliva substitutes, saliva stimulants (e.g., pilocarpine), artificial tears, vaginal lubricants, and steroids with other immunosuppressants in severe conditions [13, 14].

So far, there is no single test to diagnose pSS; however, there are different tools that are clinically applied, such as the ophthalmological examination (detects dry eyes), sialometry (assesses hyposalivation), radio sialography (measures glandular damage), ultrasound, labial salivary gland biopsy, and antinuclear antibodies profile [13]. Many studies have compared the clinical manifestations in pSS, including xerostomia, xerophthalmia, lymphoma, renal and pulmonary involvements along with the immunological profile such as the antinuclear antibodies (ANA) and rheumatoid factor (RF) and Ro/SSA [15–18]. Other studies have also assessed various blood parameters in pSS, such as complete blood count (CBC), erythrocyte sedimentation rate (ESR), and vitamins. For example, high mean platelet volume (MPV) has been detected in pSS patients, indicating disease activity [19]. Even deficiencies of vitamins such as D and B12 have been linked to pSS [20, 21].

Despite that there is no cure for pSS, various therapeutic options are clinically applied to prevent the progression of the disease based on the symptomatic and systemic manifestations [22]. Pilocarpine, artificial saliva and artificial tears, for example, are commonly used to relieve dryness of the mouth and eyes [23, 24]. In addition, the anti-malarial drug hydroxychloroquine is widely accepted in clinical practice for treating pain and decreasing extra-glandular manifestations, including pulmonary, renal, musculoskeletal, and neurological damage [25]. Immunosuppressants such as methotrexate, azathioprine or glucocorticoids like prednisone have also been suggested to improve extra-glandular symptoms in pSS [26].

pSS mainly affects females in their fourth and sixth decades of age [27]. Studies have suggested that the prevalence of pSS is around 7 times higher in elderly people [28–31], it still can still affect young individuals [16, 32]. Hence, other studies have investigated various parameters such as symptomatic manifestation (dry mouth and eyes), organ involvement, and immunological profile based on the onset of the disease [15–17, 33].

However, few laboratory features have been reported regarding the early and late onset pSS [15–18, 33]. In addition, there is a paucity of data in the literature on medication intake between these groups, specifically in Saudi Arabia. Therefore, the study aimed to investigate the prevalence and characteristics of pSS, laboratory features, and medication intake among early and late-onset pSS patients in Saudi Arabia.

## Methodology

### Study design and setting

This multicentre retrospective cohort study is based on data collection of clinically diagnosed patients with pSS at the National Guard Hospital (NGHA) electronic health record system in Saudi Arabia between 2018 and 2023. The NGHA hospitals include five hospitals: one in the central region, two in the Western region, and two in the Eastern region of Saudi Arabia. Patients with pSS were diagnosed according to the European League Against Rheumatism/American College of Rheumatology/ American College of Rheumatology (EULAR/ACR) and EULAR Sjögren’s Syndrome Disease Activity Index (ESSDAI) were considered in this study. Data of pSS patients was collected based on the International Classification of Diseases 10 (ICD 10), which is M35.0 for unspecified organ involvement and M35.09 for other organ involvement. The data was categorized into two groups: early onset, which represents patients under 40 years old, and late-onset, which represents 40 years old and above (referred to in the text as above 40). Demographic information, mortality rate, blood tests, and medications were collected accordingly [33]. Patients with any type of cancer were excluded from the study as this exploratory study focused on laboratory features and treatment management only. All patients who met the study’s criteria were included in the analysis.

### **Variables**

The age at onset variable served as our primary binary outcome. Independent variables included demographic information, including age, gender, marital status, nationality, and mortality rate. The chosen blood parameters collected were CBC, creatinine, ESR, and vitamins (primarily D and B12). In addition, the medications reported in this study were pilocarpine, artificial saliva, artificial tears, hydroxychloroquine, prednisone, methotrexate, and azathioprine.

### Ethical consideration

The study was conducted upon the approval of the Institutional Review Board (IRB) of King Abdullah Medical International Research Center under the reference number IRB/2414/23 and study number NRC23R/523/08.

### Statistical analysis

For a descriptive analysis (univariate analysis), we used the Chi-square test for categorical variables, and a t-test was employed for numeric variables. Multivariate analysis was used to examine the differences in patient characteristics using a multiple binary logistic regression model. The model assumptions of multicollinearity among independent variables have been tested. Moderate correlations (< 0.4) were observed across independent variables. A stepwise forward selection method was employed to keep only significant predictors in the final model. The analysis was performed using the SAS statistical software version 9.4. Statistical significance was determined at a *P*-value less than 0.05.

## Results

A total of 453 clinically diagnosed patients with pSS between 2018 and 2023 were included in the study, where 136 patients (30.0%) represented the early-onset group, and 317 patients (69.89%) represented the late-onset group. The mean age of the early and late onset groups was 34.2 and 60.41, respectively. The number of patients between 60 and 65 years old was 50 (11.03%), and the number of patients above the age of 65 was 45 (9.93%). Females represented the majority of pSS patients in both groups, whose marital status was predominantly married (56.6% early onset and 80.76% late onset), followed by single (34.5% under 40 and 5.0% above 40), and mainly possess Saudi nationality (96.3% under 40 and 95.5% above 40). Only 1% of patients in the early onset pSS and 5% of patients in the late-onset pSS were pronounced dead after diagnosis (Table 1).

**Table 1:** Demographic information and morality rate of pSS patients based on the early and late onset of the disease. This includes age, gender, marital status, nationality, and death status

Demographic information and morality	Early onset (< 40) *N*(%)^1^	Late onset (≥ 40) *N*(%)^1^	*P*-value ^2^
	136 (30.02)	317 (69.98)	
**Age (at diagnosis)**			< 0.0001
mean	34.27	60.41	
**Gender**			0.44
Male	11 (8.09)	33 (10.41)	
Female	125 (91.91)	284 (89.59)	
**Marital status**			< .0001^3^
Married	77 (56.62)	256 (80.76)	
Divorced	3 (2.21)	11 (3.47)	
Single	47 (34.56)	16 (5.05)	
Widowed	**-----**	21 (6.62)	
Unknown	9 (6.62)	13 (4.1)	
**Nationality**			0.71
Saudi	131 (96.32)	303 (95.58)	
Non-Saudi	5 (3.68)	14 (4.42)	
**Mortality rate**			0.02^3^
Yes	1 (0.74)	16 (5.05)	
No	135 (99.26)	301 (94.95)	

1 N(%) frequency and percntage.

2 Univariate analysis chi-square for categorical variables and t-test for numeric variables.

3 Fisher exact test.

We explored the differences in blood parameters, including CBC, creatinine, ESR, and vitamins, between both groups at diagnosis. Most blood chemistry results fell under the reference range except for hematocrit and ESR levels. Haematocrit was slightly under the normal range in females (0.37–0.47 L/L) in the early onset at a value of 0.36 L/L but was within the normal range in the late-onset (*P* = 0.001), given that the majority of patients were females. However, the difference between both groups’ hematocrit values was statistically significant (*P* = 0.001). In addition, a considerable increase in ESR was detected in early and late-onset pSS patients (46.3 mm/hr in the early onset and 49.8 in the late onset). It should be emphasized that vitamin D levels were normal, yet values among the early onset (57.2 nmol/L) and late-onset (68.6 nmol/L) were in the lower limit of the normal range, and a significant difference in vitamin D levels was identified between both groups (*P* = 0.009) (Table 2).

**Table 2:** Blood parameters of pSS at the early and late onset of the disease. This comprised CBC, creatinine, ESR, and vitamins presented as mean and standard deviation (SD±).

Blood parameters	Early onset (< 40) *N*(%)^1^	Late onset (≥ 40) *N*(%)^1^	*P*-value ^2^
	136 (30.02)	317 (69.98)	
**Hemoglobin (Hb)** (Male: 130 to 180 g/L, Female: 115–165 g/L)			0.29
Mean	125±14.6	128.5±14.84	
**Haematocrit (Hct)** (Male: 0.40–0.54 L/L, Female: 0.37–0.47 L/L)			0.001
Mean	0.36±2.58	0.38±0.048	
**MCH** (27 to 32 pg)			0.82
Mean	27.82±0.04	27.9±2.53	
**MCV** (80–100 fl.)			0.29
Mean	85.78±7.66	86.92±7.54	
**MPV** (8–12 fL)			0.72
Mean	8.49±1.14	8.43±1.12	
**NRBC#** (< 0.3)			0.55
Mean	0.0024±0.01	0.005±0.05	
**NRBC %**			0.58
Mean	0.03±0.12	0.06±0.76	
**Platelet Count** (140–400 × 10^9^ /L)			0.02
Mean	308.4±93.15	286.4±81.52	
**Red blood cells (RBC)** (Male: 4.5–6.5 × 10^12^/L, Female: 3.8–5.80 × 10^12^/L)			0.08
Mean	4.32±0.49	4.49±0.54	
**Red cell distribution (RDW)** (11.5–15.7%)			0.46
Mean	13.88±1.70	13.71±1.74	
**White Blood Cell Count (WBC)** (3.60–11.0 × 10^9^ /L			0.7564
Mean	6.63±2.50	6.52±2.17	
**Creatinine** (Male: 59–104 µmol/L, Female:45–84 µmol/L)			0.001
Mean	62.73±18.46	74.46±50.36	
**Sedimentation Rate (ESR)** (Females < 50 years: ≤20 mm/hr, Females > 50 years old: ≤30 mm/hr)			0.55
Mean	46.36±28.64	49.85±25.88	
**VIT D 25 OH (50–125 nmol/L)**			0.009
Mean	57.23±28.40	68.68±25.23	
**Vitamin B12 (180–1000 pg/mL)**			0.05
Mean	298.4±132.95	346.5±195.54	

1 N(%) frequency and percentage.

2 Univeriate analysis chi-aqure for categorical variables and t-test for numeric variables.

The list of medications prescribed to pSS patients (Table 3), shows a significant difference in the type of medication intake among early and late-onset pSS patients. For example, less than 22% (*P* = 0.68) of patients used pilocarpine, and less than 6% (*P* = 0.16) used artificial saliva, with comparable patterns of medication use among both groups. In contrast, artificial tears were highly prescribed to late-onset compared (72.6%) to the early-onset group (52.9%) (*P* = 0.0001). Interestingly, hydroxychloroquine was the only predominant medication in both early (78.1%) and late-onset (68.5%) (*P* = 0.0001). Moreover, approximately 50% of early-onset pSS patients received prednisone, whereas only 38.7% of late-onset pSS patients received 30.88% of the same drug, presenting a significant difference among both groups (*P* = 0.03). In contrast, a low number of patients, regardless of their age, used the immunosuppressants methotrexate (*P* = 0.05) and azathioprine (*P* = 0.04) (Table 3).

**Table 3:** The frequency of medication prescribed to pSS patients is based on the early and late onset of the disease

Medication	Early onset (< 40) *N*(%)^1^	Late onset (≥ 40) *N*(%)^1^	*P*-value^2^
	136 (30.02)	317 (69.98)	
**Pilocarpine**			0.68
Yes	23 **(**19.33)	61 (21.11)	
No	96 **(**80.67)	228 (78.89)	
**Artificial saliva** ^**3**^			0.16
Yes	3 (2.21)	16 (5.05)	
No	133 (97.79)	301 (94.95)	
**Artificial tears** ^**3**^			0.0001
Yes	63 (52.94)	210 (72.66)	
No	56 (47.06)	79 (27.34)	
**Hydroxychloroquine** ^**3**^			0.05
Yes	93 (78.15)	198 (68.51)	
No	26 (21.85)	91 **(**31.49)	
**Prednisone** ^**3**^			0.03
Yes	60 **(**50.42)	112 **(**38.75)	
No	59 (49.58)	177 **(**61.25)	
**Methotrexate** ^**3**^			0.0545
Yes	11 (9.24)	48 **(**16.61)	
No	108 (90.76)	241 **(**83.39)	
**Azathioprine** ^**3**^			0.004
Yes	21 (17.65)	23 (7.96)	
No	98 (82.35)	266 (92.04)	

1 N(%)frequency and percentage.

2 Univariate analysis Chi-square for categorical variables and t-test for numeric variables.

3 Missing values.

The regression analysis was performed to predict the probability of encountering different parameters during early onset pSS. For instance, married patients were less likely to be diagnosed with early onset compared to non-married patients, with an odds ratio (OR) of 0.33, 95% confidence interval (CI) (0.18, 0.5), *P* < 0.0001, and less likely to be non-Saudi (OR 0.43; CI (1.06, 71.89), *P* = 0.23. Patients who did not receive artificial tears were more than two times more likely to be diagnosed with early onset than those who did receive artificial tears (OR 2.12; CI (1.31, 3.42), *P* = 0.0003. Patients who did not receive hydroxychloroquine (OR 0.66; CI (0.38, 1.14), *P* = 0.12, prednisone (OR 0.52; CI (0.31, 0.88), *P* = 0.07, and methotrexate (OR 0.42; CI (0.21, 0.86), *P* = 0.04, were less likely to be diagnosed with early onset compared to those who did receive these medications (Table 4).

**Table 4:** Multivariate logistic regression analysis for predictors of early onset

	Early vs. Late onset Odd ratio	95% Confidence interval	*P*-value
**Marital status**			
Married	0.31	(0.18, 0.5)	< 0.0001
Not-Married	Reference group	Reference group	
**Nationality**			
Saudi	Reference group	Reference group	
Non-Saudi	0.43	(0.11, 1.75)	0.23
**Mortality**			
Yes	Reference group	Reference group	
No	8.76	(1.06, 71.89)	0.02
**Artificial tears**			
Yes	Reference group	Reference group	
No	2.12	(1.31, 3.42)	0.0003
**Hydroxychloroquine**			
Yes	Reference group	Reference group	
No	0.66	(0.38, 1.14)	0.12
**Prednisone**			
Yes	Reference group	Reference group	
No	0.52	(0.31,0.88)	0.07
**Methotrexate**			
Yes	Reference group	Reference group	
No	0.42	(0.21, 0.86)	0.04

## Discussion

Primary Sjogren syndrome is a heterogeneous disorder characterized by glandular and extra-glandular manifestations. Clinical, histological, immunological, and subjective symptoms (dry mouth and eyes) can all assist in diagnosing pSS [34]. There is no cure for pSS, but there is a variety of therapeutic options that could ameliorate symptoms [13]. It has been reported that the prognosis of autoimmune diseases can be affected by age at the onset of the disease and clinical findings [35–37]. Therefore, this study aimed to explore the laboratory findings and treatment management among pSS based on the onset of the disease in Saudi Arabia.

It has been previously documented that pSS mainly affects individuals between the ages of 40 and 60 years old [27]. Hence, in this study, we considered the cut-off point of the onset of the disease to be under 40 years, despite other studies defining early onset at or under the age of 35 [16, 18, 38]. The total number of patients with pSS at or under 35 years old was 11.4% in the Turkish population and 13.9% (55 out of 393 patients) in the French population [16, 33]. On the other hand, our findings show that the mean age of patients under 40 was 34.27, which accounts for 30% of this study population. This raises the question of whether the development of pSS was influenced by geoepidemiology or ethnicity, as these factors have been associated with pSS incidence in other studies [39, 40].

Our findings show that the prevalence of pSS in females is higher than in males, which alighns with findings from other studies [6]. Additionally, the main proportion of these patients were married. It has been suggested that troubled relationships could contribute to stress and depression, which in turn dysregulate the immune system [41]. Another study indicated that the long-term accumulation of physiological stress, even before developing pSS, can increase the likelihood of developing such an autoimmune disease [42]. However, pSS is defined as a chronic, non-life-threatening disease, and organ-specific manifestations that could arise upon or after diagnosis can impact SS prognosis. According to recent studies, most of the pSS-related mortality is not associated with systemic autoimmune damage nor directly with systemic pSS [3, 4]. Instead, the high risk of morality has been associated with extra glandular involvement, vasculitis, hypocomplementaemia, and cardiovascular diseases [43, 44]. Yayla et al. detected around 5% of deaths above the age of 35 [33], and a corresponding finding was also observed in this study. However, it is difficult to identify the definite causes of death at this stage. Exploring other systemic manifestations would probably provide a better understanding of the causes of death.

Abnormal blood parameters have been previously detected in pSS. For instance, a study reported a reduction in platelet count and white blood cells and an increase in ESR levels up to 50 mm/h, besides renal involvement and peripheral neuropathy in pSS patients [45]. A noticeable elevation of ESR, red cell distribution width (RDW), and C-reactive protein (CRP) have been associated with pSS, which could reflect disease activity [46–48]. Herein, we noticed a slight decrease in hematocrit but a significant elevation of ESR. It has been reported that abnormal ESR is affected by age [49]; however, this abnormality was observed even in patients under 40 years old, which aligns with findings from other studies on pSS patients [45, 50].

Furthermore, creatinine was chosen in this study to interpret the level of renal involvement in pSS patients. It has been suggested that creatinine could be a valuable biomarker if combined with alpha one macroglobulin (α1-MG) rather than alone for diagnosing renal diseases [51, 52]. However, further investigation is required as creatinine alone is insufficient to identify the renal involvement, knowing that elevated creatinine is common in the elderly population [53].

In the context of vitamin deficiencies, a systemic review and meta-analysis of 18 studies highlighted the role of vitamin D deficiency in pSS and the severity of dry eyes [20]. Although the data presented in this study showed normal vitamin D levels either in patients under or above 40 years, it is worth noting that the values of both groups fall near the border of the normal range, suggesting a possible drop that could arise if patients are not consuming adequate supplements or do not receive sufficient exposure to sunlight. Studies have shown that one-third of the Middle Eastern population, and particularly 40% of Saudis aged between 15 and 49, have anemia as a result of iron and vitamin B12 deficiencies [54, 55]. The fact that normal B12 and CBC were detected in this study suggests that anemia is not a feature of pSS. However, iron and ferritin assessments would help confirm this observation.

To our knowledge, only one study compared the treatment regimen used in pSS, such as pilocarpine, hydroxychloroquine, and methotrexate between the early and late onset pSS patients [33]. However, this is the first study that provided insight into the medication intake between early and late-onset among Saudi population. Treatment strategies for systemic manifestation depend on the clinical signs and disease activity [24]. The parasympathomimetic drug pilocarpine stimulates salivary gland function through the muscarinic M3 receptor and the parasympathetic system (when given orally) [56]. Nevertheless, pilocarpine may induce side effects as it affects these muscarinic receptors located in most organ systems; this includes excessive sweating and urination, stomach upset and dizziness [57, 58]. This might explain why pilocarpine was unpopular in most of the study population. Artificial saliva is another example of a saliva replacement that moisturizes oral tissues and is available in diverse forms, such as liquids, gels, sprays, and mouthwash [59]. It has been reported that using artificial saliva as a palliative or coadjuvant therapy depends on the severity of dry mouth [60]. The minimal use of artificial saliva could suggest that dry mouth was not the main complaint in pSS patients or that other alternatives to artificial saliva were prescribed to these patients. Artificial tears are the first-line treatment for dry eye diseases, including pSS [61]. Patients above 40 years were prescribed artificial tears more than those under 40. The rationale behind this is that eye dryness in SS is expected to worsens with chronic diseases and age [62].

Hydroxychloroquine is one of several available therapeutic options that exhibit good safety with minimal side effects. It protects against systemic damage and improves ocular symptoms and laboratory parameters, such as ESR, CRP, immunoglobulin M (IgM), and immunoglobulin A (IgA) [63]. This explains why hydroxychloroquine was placed as the first-line therapy compared to other treatment regimens among early and late-onset pSS. Also, prednisone has been shown to improve Sicca syndrome and decrease IgA, IgG, and ESR [64]. However, it has been suggested that even low doses of prednisone for less than 18 months can predispose to osteoporosis [65]. Since patients older than 40 years old use this medication, it raises a concern about the likelihood of developing osteoporosis, given that osteoporosis is prevalent in Saudi females aged 50 or above [66]. It has been reported that methotrexate is useful for pain and joint inflammation in pSS [67, 68]. In addition, methotrexate is usually prescribed as a second-line therapy after hydroxychloroquine [69]. The high number of patients who did not receive methotrexate possibly implies that these populations either did not tolerate the drug or experienced difficulty with hydroxychloroquine. We also noticed that some patients, either under or above the age of 40, have been prescribed azathioprine. Studies have reported that myelosuppression and hepatotoxicity are the main adverse effects of azathioprine, which acts in a dose and duration-dependent manner. Intolerance to azathioprine could justify the low number of patients receiving this drug [70, 71].

## Limitations

Data presented in this study provided insight into the frequency of pSS in Saudi Arabia and the laboratory characteristics of pSS. However, including symptomatic and systemic manifestations such as dry mouth, dry eyes, arthralgia, arthritis, average lymphocyte count, CRP, pulmonary involvement, and renal involvement, together with immunologic features like ANA and anti-Rho/SS-A, would support these findings. In addition, the incorporation of the diagnostic criteria of SS EULAR/ACR to describe the characteristics of pSS patients and the interpretation of our findings in parallel with the EULAR Sjögren’s syndrome disease activity index (ESSDAI) would enhance the value of this study. However, the lack of this important factor in the electronic health system prevented us from including them in this study. Thus, a potential confounding bias is expected. Another limitation of the study is associated with the nature of such an observational study. As a retrospective study, we cannot determine causation; only association can be drawn from the conclusion of the study.

## Conclusion

This study shed light on the clinical characteristics of early and late-onset pSS amongst Saudi patients. The incidence of pSS in young individuals drew attention to the fact that the susceptibility of pSS is not restricted to 40–60 years old in the Saudi population. Although blood profiles did not show significant differences among these patients, ESR appeared abnormal; others could indicate a potential decrease in the future as their levels are at the border of the normal range, such as hematocrit and vitamin D. In addition, the variation in medication intake between early and late-onset could indirectly indicate the severity of pSS.

## Data Availability

The data supporting this study’s findings are available from the corresponding author upon reasonable request.
